# The emerging picture of a complex core-mantle boundary

**DOI:** 10.1038/s41467-024-48939-1

**Published:** 2024-05-29

**Authors:** Stuart Russell, Jessica C. E. Irving, Robert Myhill, Sanne Cottaar

**Affiliations:** 1 University of Cambridge, Bullard Laboratories, Madingley Road, Cambridge, CB3 0EZ UK; 2 Universität Münster, Institut für Geophysik, Corrensstraße 24, 48149 Münster, Germany; 3 University of Bristol, School of Earth Sciences, Wills Memorial Building, Bristol, BS8 1RL UK

**Keywords:** Geophysics, Geodynamics, Geochemistry, Core processes, Seismology

## Abstract

Recent seismological studies challenge the traditional view that the interface between the core and mantle is a straightforward discontinuity. As seismology is pushed to its observational limits, a complex - potentially compositionally layered - region between the core and mantle is emerging.

## The core-mantle boundary

Earth comprises a series of approximately spherical shells, each with their own properties and character; these are separated by discontinuities where the physical and/or chemical properties are assumed to change abruptly. The most extreme of these discontinuities is the core-mantle boundary (CMB), where, at a depth of almost 2900 km below the surface, the rocky mantle meets the iron core. Below the CMB, the liquid metal outer core convects vigorously, generating Earth’s geomagnetic field. Above the boundary, the solid mantle also convects, albeit with much smaller characteristic velocities, the surface expression of which is plate tectonics. Both of these convective processes are controlled, at least in part, by heat flux across the CMB; this boundary, therefore, plays a vital role in controlling planetary-scale processes that directly affect the evolution and habitability of our planet.

In many global seismological models of the Earth, the CMB is considered to be a sharp discontinuity between the solid silicate mantle and the liquid iron core [e.g. ^1^]. The density increase across the CMB exceeds 4 g/cm^3^, which is greater than the contrast between rock and air at Earth’s surface. This large density contrast raises the possibility that material of intermediate density could be trapped at the interface between the two regions, complicating the CMB relative to the global models. This material may be swept by, but not wholly entrained in, bulk mantle flow^2^ and able to influence heat flow and chemical exchange across the CMB.

In some places, material trapped at the CMB has been observed seismically: ultra-low velocity zones (ULVZs) are regions of extremely reduced seismic velocity sitting atop the CMB that are typically tens of kilometers thick. The seismic velocities in ULVZs are typically 10–50% lower than in the background mantle, with absolute shear wave velocities as low as 3.5 km s^*−*1^. Whether ULVZs are isolated patches of material or thickened regions of an otherwise global layer remains unclear. A global layer, if it were too thin, may not be readily detectable by seismologists–a minimum detectable thickness of 5 km has been suggested^3^. While various studies favour particular formation mechanisms, there is no firm consensus on the origin and nature of ULVZs, and the wide range of observed properties indicates that they may have a range of origins. Here, we reflect on observations and arguments that indicate the core-mantle boundary may be complex on the scale of ULVZs and smaller.

## Material trapped at the core-mantle boundary

There are several mechanisms that could cause compositionally distinct material to accumulate at the CMB and contribute to the formation of a global layer or ULVZ-like patches of material. Mantle-derived material could include the remnants of primordial heterogeneities left over from the deep fractional crystallisation of an ancient magma ocean formed during Earth’s formation^4^. Other possibilities include the remains of near-surface igneous or sedimentary rocks introduced into the lowermost mantle via deep subduction^5,6^. These near-surface rocks may partially melt at the temperatures and pressures of the CMB resulting in the accumulation of dense, iron-rich melt above the boundary^2^.

In the core, the tendency of light elements to migrate to lower pressures during barodiffusion could cause light-element-rich liquid material to accumulate below the CMB^7^. An alternative mechanism to generate light-element-rich liquid is the early exsolution of Mg- and Si-rich liquid from the core^8^. These mechanisms could cause layering near the CMB. However, major uncertainties remain as to the extent to which these processes have occurred in the Earth, the preservation potential of such compositional layering, and the seismic visibility and characteristics of such layers^9^.

Solid phases, which would rise due to being less dense than the iron liquid, could also precipitate as the core cools and “snow” upwards onto the CMB. The mineral phases periclase (MgO), seifertite (SiO_2_), and FeSi have all been proposed as equilibrium snow components at different times in Earth’s history^9–11^. The presence and identity of a snowing phase depends on the bulk composition of the liquid outermost core and on the phase diagram of iron alloys under outermost core conditions, neither of which are well constrained at present.

Bulk core and mantle material are thought not to be in chemical equilibrium and should therefore react^12^. These reactions could create and/or modify material across the boundary. For example, core-derived seifertite and mantle-derived ferropericlase would react to form bridgmanite. Core-derived MgO-rich ferropericlase or melt would undergo Mg-Fe exchange with more FeO-rich mantle material. Finally, an ancient mantle-derived FeO-rich layer at the base of the mantle would be likely to add oxygen to the core. The thickness and stability of the compositionally distinct layer that results from these different reactions would depend on the dynamics and chemical diffusivities of the materials at the CMB.

## Seismic detection of a layered core-mantle boundary

Several recent studies have made progress towards resolving small-scale heterogeneities close to the CMB. As more of the CMB has been explored, ULVZ detections have become more commonplace [e.g. ^3,13^]. One recent study^5^ used core-reflected PcP waves to probe the CMB beneath Antarctica. They found their study region is underlain by ubiquitous ULVZs, of variable thickness and properties, which is intriguing given that the CMB beneath Antarctica is far removed from present-day mantle up- and down-wellings. Using geodynamic modelling, they demonstrated that their observations were consistent with the settling of dense subducted material into a global layer at the CMB. Furthermore, there are now observations of internal kilometre-scale layering in several ULVZs with greater velocity reductions in the basal layer^14,15^.

Beneath the central Pacific, observations of CMB-diffracted shear waves (S_diff_) were used to demonstrate that an extensive region may be entirely underlain by a thin and slow layer atop the CMB^16^. The study prefers a layer that is 5 km thick with a 14% shear velocity reduction but notes a substantial trade-off between layer thickness and velocity reduction.

Another study^17^ directly addressed the possibility of a globally layered CMB. Using synthetic waveform modelling, they demonstrated that a previously underutilised diffracted phase, PKKP_diff_, was very sensitive to layering, even when only a kilometre or so thick. Assembling a dataset of over 12,500 PKKP_diff_ observations, they found their travel-time data exhibited an offset relative to expected values that was consistent with a global, seismically slow layer above the CMB that is approximately 1–2 km thick. However substantial scatter in the data, which could itself be due to complexity in the lowermost mantle, and the dependence on choice of background model prevented conclusive identification of such a layer.

All the research discussed above has used body waves – relatively short-period waves that propagate outwards from an earthquake source. In contrast, normal modes are extremely long-period whole-Earth oscillations observed after very large earthquakes. The frequencies that normal modes oscillate at provide constraints on Earth’s internal structure. Recent work suggests that normal mode centre frequency observations are consistent with a dense and slow kilometre-scale layer atop the CMB^18^.

## Multi-disciplinary indicators of a complex core-mantle boundary

Important additional constraints on CMB structure come from a variety of geophysical disciplines. Geodetic measurements of Earth’s rotation indicate that there is a transfer of momentum from the core to the mantle. The favoured mechanism to achieve this transfer is electromagnetic coupling, whereby the geomagnetic field induces currents in the lowermost mantle which, in turn, cause a torque [e.g. ^19,20^]. If this mechanism were solely responsible for the coupling, the lowermost mantle would need to have an electrical conductivity on the order of 10^5^ Sm^−1^. This is several orders of magnitude higher than is expected for silicates at CMB conditions^21^. A thin, global layer of iron-rich material could satisfy both the coupling and the seismic observations as the conductivity of FeO at the CMB is on the order of 10^4^ Sm^−1^ − 10^5^ Sm^−1^ ^22,23^. Such a layer would also be dense and seismically slow^24^.

**Fig. 1:**
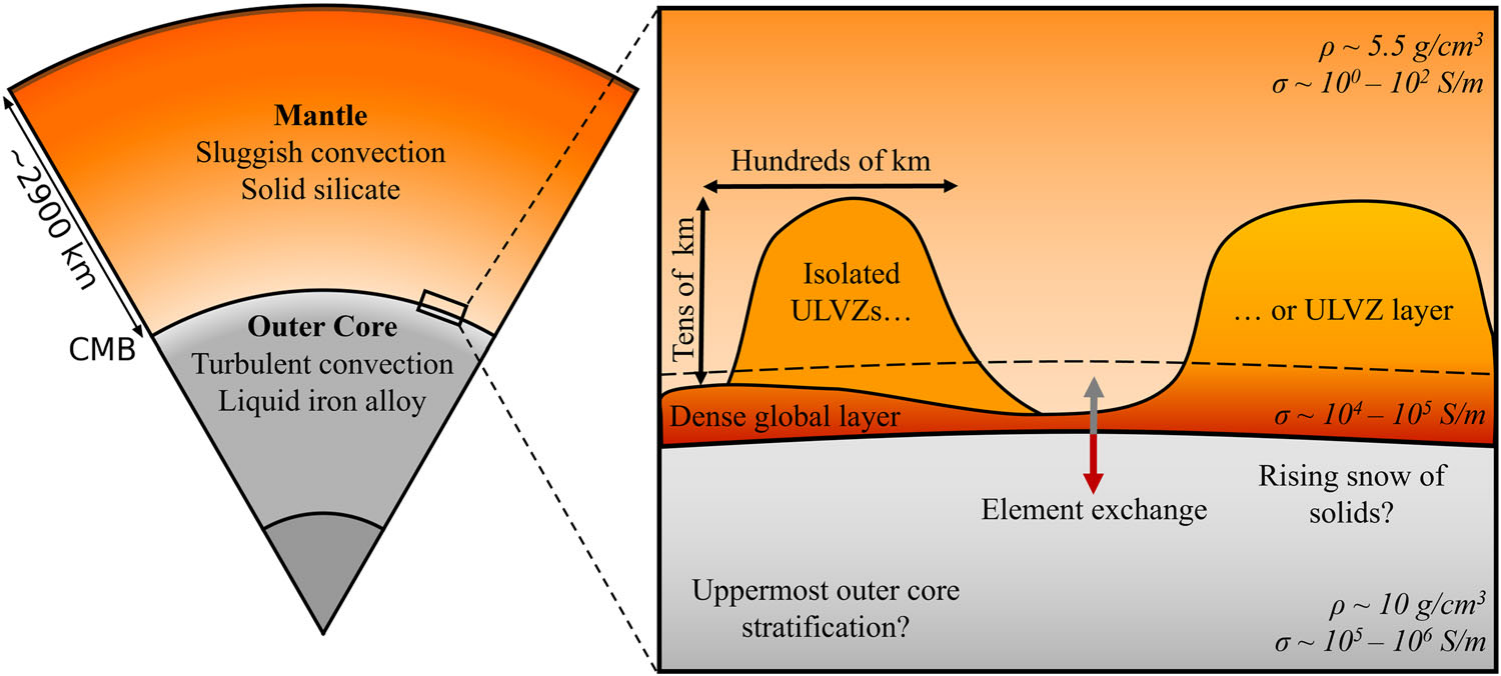
Cartoon showing the place of a complex CMB in the Earth system (left) and a zoom-in of a possible complex CMB scenario and the relationship of a basal layer to ULVZs (right). The dashed line indicates an estimated limit of robust body wave detection, which lies above the average layer thickness. Density and electrical conductivity values are denoted by *ρ* and *σ*, respectively.

A chemically reactive iron- or melt-rich base to the mantle could also help to satisfy geochemical observations related to core-mantle interaction [e.g. ^25^]. For example, such a layer may facilitate the formation of stratification beneath the CMB by altering the ratios of light elements in the outermost core [e.g. ^9,26^]. Partial entrainment of a layer by mantle convection may lead to geochemical signatures measured at hotspots, including anomalous helium and tungsten ratios, which are interpreted to be tracers of core-mantle interaction^27,28^. Lateral variations in CMB-associated layering may influence the structure and temporal variation of the geomagnetic field by affecting CMB heat flux^29^. Figure 1 depicts possible structure at the CMB and relevant properties and processes.

## Outlook

Despite the CMB being the subject of nearly a century of research^30^, recent scientific advances force us to re-evaluate our understanding of this planetarily-important boundary. Recent results from seismology highlight the heterogeneous nature of the CMB, providing a growing body of evidence for a complex, potentially layered zone at the base of the silicate mantle. The existence of a highly conductive layer is also supported by geodetic and geomagnetic constraints, indicating that the lowermost mantle above the CMB may be iron-enriched.

Even with these advances, seismological observations have not yet unequivocally resolved global layering at the CMB. A concerted multi-disciplinary effort will be required to better constrain the presence and characteristics of this layering. This work could help us understand temporal and lateral variations in CMB heat flux, and thus the dynamics of the outer core and the nature of the geomagnetic field. In addition, further experimental, thermodynamic, geochemical, and geodynamic work will be needed to understand the chemical and dynamic interaction between solid or fluid layers and mantle and core materials, and the evolution of these interactions over Earth’s history. Given the number of ways in which CMB structure can affect Earth observations, it is exciting to see novel studies that are revealing more of its intriguing complexity.

## References

[1] Kennett B (2020). Radial earth models revisited. Geophys. J. Int..

[2] Dannberg J, Myhill R, Gassmöller R, Cottaar S (2021). The morphology, evolution and seismic visibility of partial melt at the core-mantle boundary: Implications for ULVZs. Geophys. J. Int..

[3] Yu S, Garnero EJ (2018). Ultralow velocity zone locations: A global assessment. Geochem. Geophys. Geosyst..

[4] Labrosse S, Hernlund JW, Coltice N (2007). A crystallizing dense magma ocean at the base of the Earth’s mantle. Nature.

[5] Hansen SE, Garnero EJ, Li M, Shim S-H, Rost S (2023). Globally distributed subducted materials along the Earth’s core-mantle boundary: Implications for ultralow velocity zones. Sci. Adv..

[6] Keller, D. S. et al. Links between large igneous province volcanism and subducted iron formations. *Nat. Geosci.* **16**, 527–533 (2023).

[7] Gubbins D, Davies CJ (2013). The stratified layer at the core–mantle boundary caused by barodiffusion of oxygen, sulphur and silicon. Phys. Earth Planet. Inter..

[8] Helffrich G, Hirose K, Nomura R (2020). Thermodynamical modeling of liquid Fe-Si-Mg-O: Molten magnesium silicate release from the core. Geophys. Res. Lett..

[9] Brodholt J, Badro J (2017). Composition of the low seismic velocity E’ layer at the top of Earth’s core. Geophys. Res. Lett..

[10] Badro J (2018). Magnesium partitioning between earth’s mantle and core and its potential to drive an early exsolution geodynamo. Geophys. Res. Lett..

[11] Fu S, Chariton S, Prakapenka VB, Shim S-H (2023). Core origin of seismic velocity anomalies at Earth’s core–mantle boundary. Nature.

[12] Trønnes R (2019). Core formation, mantle differentiation and core-mantle interaction within Earth and the terrestrial planets. Tectonophysics.

[13] Thorne MS (2021). The most parsimonious ultralow-velocity zone distribution from highly anomalous SPdKS waveforms. Geochem. Geophys. Geosyst..

[14] Ross, A. R., Thybo, H. & Solidilov, L. N. Reflection seismic profiles of the core-mantle boundary. *J. Geophys. Rese.: Solid Earth* **109**, B08303 (2004).

[15] Li Z, Leng K, Jenkins J, Cottaar S (2022). Kilometer-scale structure on the core–mantle boundary near Hawaii. Nat. Commun..

[16] Wolf J, Long MD (2023). Lowermost mantle structure beneath the central Pacific Ocean: Ultralow velocity zones and seismic anisotropy. Geochem. Geophys. Geosyst..

[17] Russell S, Irving JCE, Cottaar S (2022). Seismic visibility of melt at the core-mantle boundary from PKKP diffracted waves. Earth Planet. Sci. Lett..

[18] Russell S, Irving JCE, Jagt L, Cottaar S (2023). Evidence for a Kilometer-Scale Seismically Slow Layer Atop the Core-Mantle Boundary From Normal Modes. Geophys. Res. Lett..

[19] Buffett BA, Mathews PM, Herring TA (2002). Modeling of nutation and precession: effects of electromagnetic coupling. J. Geophys. Res.: Solid Earth.

[20] Holme R, De Viron O (2013). Characterization and implications of intradecadal variations in length of day. Nature.

[21] Sinmyo R, Pesce G, Greenberg E, McCammon C, Dubrovinsky L (2014). Lower mantle electrical conductivity based on measurements of Al, Fe-bearing perovskite under lower mantle conditions. Earth Planet. Sci. Lett..

[22] Holmström E, Stixrude L, Scipioni R, Foster AS (2018). Electronic conductivity of solid and liquid (Mg, Fe)O computed from first principles. Earth Planet. Sci. Lett..

[23] Ho, W.-G. D. et al. Quantum critical phase of FeO spans conditions of Earth’s lower mantle. *Nat. Commun.* **15**, 3461 (2024). 10.1038/s41467-024-47489-w PMC11043421 38658590

[24] Wicks JK, Jackson JM, Sturhahn W (2010). Very low sound velocities in iron-rich (Mg, Fe)O: Implications for the core-mantle boundary region. Geophys. Res. Lett..

[25] Lim KW, Bonati I, Hernlund JW (2021). A hybrid mechanism for enhanced core-mantle boundary chemical interaction. Geophys. Res. Lett..

[26] Waszek L, Irving JCE, Pham T-S, Tkalčić H (2023). Seismic insights into Earth’s core. Nat. Commun..

[27] Mundl-Petermeier A (2020). Anomalous 182W in high 3He/4He ocean island basalts: Fingerprints of Earth’s core?. Geochim. et. Cosmochim. Acta.

[28] Horton F (2023). Highest terrestial 3He/4He credibly from the core. Nature.

[29] Mound JE, Davies CJ (2023). Longitudinal structure of Earth’s magnetic field controlled by lower mantle heat flow. Nat. Geosci..

[30] Jeffreys H (1926). The Rigidity of the Earth’s Central Core. Geophys. Suppl. Month. Not. R. Astron. Soc..

